# Atherosclerotic Plaque Destabilization in Mice: A Comparative Study

**DOI:** 10.1371/journal.pone.0141019

**Published:** 2015-10-22

**Authors:** Helene Hartwig, Carlos Silvestre-Roig, Jeffrey Hendrikse, Linda Beckers, Nicole Paulin, Kim Van der Heiden, Quinte Braster, Maik Drechsler, Mat J. Daemen, Esther Lutgens, Oliver Soehnlein

**Affiliations:** 1 Department of Pathology, Academic Medical Center, Amsterdam, The Netherlands; 2 Institute for Cardiovascular Prevention (IPEK), LMU Munich, Munich, Germany; 3 Department of Medical Biochemistry, Academic Medical Center, Amsterdam, The Netherlands; 4 Department of Cardiology, Biomedical Engineering, Erasmus MC, Rotterdam, The Netherlands; 5 German Centre for Cardiovascular Research (DZHK), Munich Heart Alliance, Munich, Germany; Monash University, AUSTRALIA

## Abstract

Atherosclerosis-associated diseases are the main cause of mortality and morbidity in western societies. The progression of atherosclerosis is a dynamic process evolving from early to advanced lesions that may become rupture-prone vulnerable plaques. Acute coronary syndromes are the clinical manifestation of life-threatening thrombotic events associated with high-risk vulnerable plaques. Hyperlipidemic mouse models have been extensively used in studying the mechanisms controlling initiation and progression of atherosclerosis. However, the understanding of mechanisms leading to atherosclerotic plaque destabilization has been hampered by the lack of proper animal models mimicking this process. Although various mouse models generate atherosclerotic plaques with histological features of human advanced lesions, a consensus model to study atherosclerotic plaque destabilization is still lacking. Hence, we studied the degree and features of plaque vulnerability in different mouse models of atherosclerotic plaque destabilization and find that the model based on the placement of a shear stress modifier in combination with hypercholesterolemia represent with high incidence the most human like lesions compared to the other models.

## Introduction

Acute coronary syndromes (ACS) are the main cause of morbidity and mortality in western societies. Thrombotic events associated to plaque rupture are responsible of the majority of ACS events [1, 2]. Rupture-prone plaques are archetypically characterized by exacerbated infiltration of inflammatory leukocytes in combination with a large necrotic core (NC) covered by a thin fibrous cap (FC) [3]. During plaque destabilization, enhanced macrophage and vascular smooth muscle cell (VSMC) apoptosis fuel the NC enlargement. In addition, extracellular matrix degradation and VSMC apoptosis induction by lesional proteases results in the loss of the FC stability [4]. Structural damage in the FC results in the exposure of the highly thrombogenic material of the NC to the blood, leading to thrombus formation, occlusion of the coronary artery, and associated ACS [5]. Albeit the clinical relevance of this process, the understanding of the mechanisms underlying the conversion of a stable into an unstable and ruptured plaque remains unclear.

Various mouse models have been proposed in the last decades to study the process of atherosclerotic plaque destabilization. Generation of vulnerable plaques has been achieved by diverse strategies including genetic modifications, surgical techniques or combination of both in genetically-modified hypercholesterolemic mouse models (reviewed in [4]). Murine vulnerable plaques resemble some features of advanced human plaques such as large NC, thin FC and the presence of intraplaque hemorrhages or neoangiogenesis. On the other hand, plaque rupture and associated thrombotic events are almost never detected. Besides these differences between murine and human vulnerable lesions, mouse models may be highly helpful in the study of the pathomechanisms that lead to vulnerable plaques prior to rupture [5, 6]. However, a consensus model of mouse atherosclerotic plaque destabilization is still lacking.

In this study, we aimed to evaluate the degree of plaque vulnerability in different mouse model of atherosclerotic plaque destabilization. [7–9]. We employed two models in apolipoprotein E deficient (*Apoe* ^*-/-*^) mice of local shear stress alteration based on the partial ligation of and/or the implantation of a shear stress modifier device in the left common carotid artery were employed. In addition, we induced endogenous renovascular hypertension through partial ligation of the left renal artery. Finally, we examined the effect of aggravated hypercholesterolemia on plaque development in these models by feeding the mice chow or high fat diet. In conclusion, we observed that the model based on the implantation of a shear stress modifier device under hypercholesterolemia exhibited higher incidence of human-like vulnerable atherosclerotic plaques formation and reduced variability between specimens.

## Materials and Methods

### Ethics statement

This study was carried out in strict accordance with the recommendations in the Guide for the Care and Use of Laboratory Animals of the National Institutes of Health. The protocol was approved by the Committee on the Ethics of Animal Experiments of the University of Amsterdam and the Animal Research Institute (ARIA), Amsterdam (Permit Number: DBC102939 and DBC102940). All animal experiments were conducted in the animal facility of the Academic Medical Center (AMC, Amsterdam). All surgeries were performed under isoflurane anesthesia, and all efforts were made to minimize suffering.

### Mice

**Fig 1:**
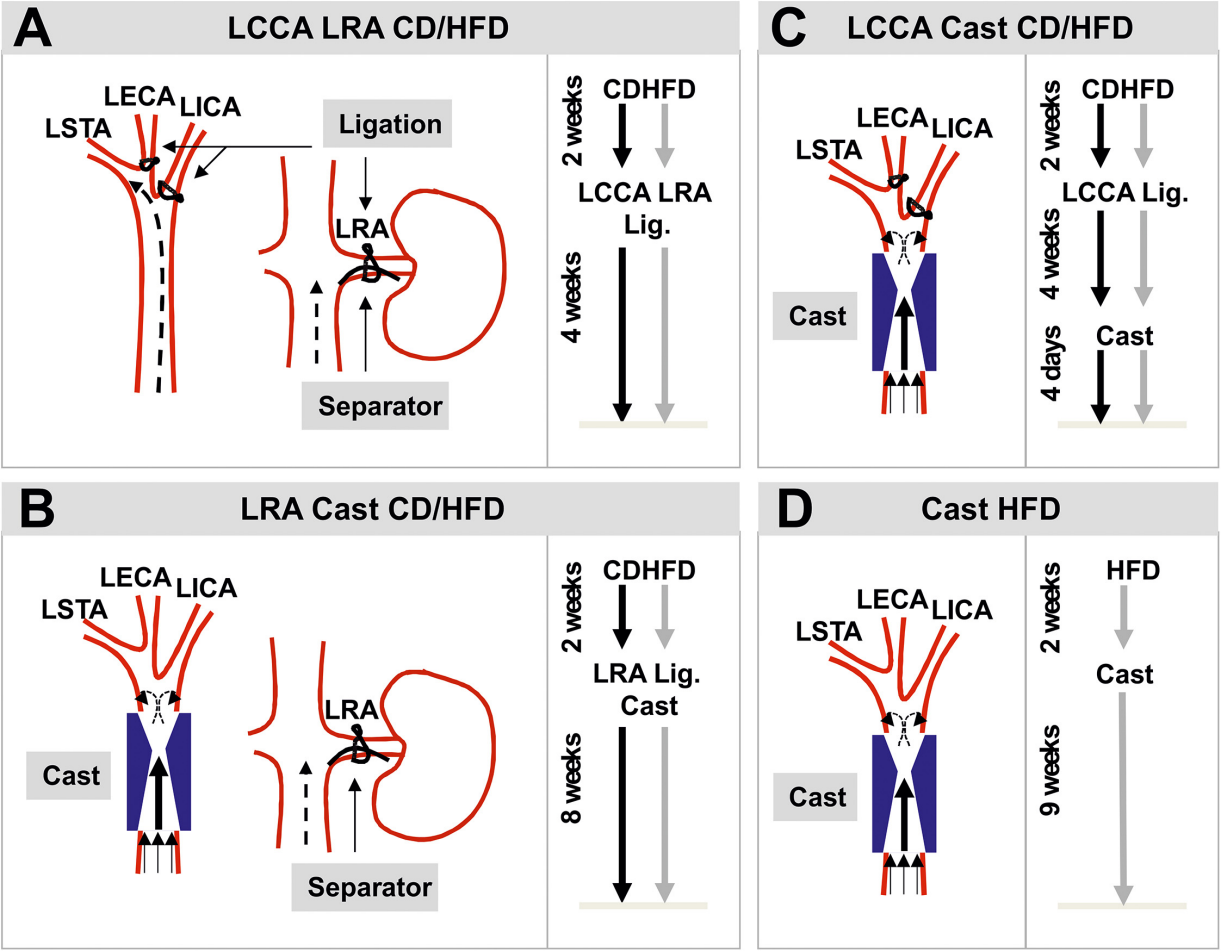
Schematic diagrams and experimental setups of the mouse models of atherosclerotic plaque destabilization. (**A**) Model based on the combined partial ligation of LCCA (left) and LRA (right). (**B**) Model based on the combination of cast placement around LCCA (left) and partial ligation of LRA (right). (**C**) Model based on the partial ligation of LCCA in combination with the cast placement around the LCCA. (**D**) Model based on cast placement around the LCCA. LCCA: left common carotid artery; LRA: left renal artery; LECA: left external carotid artery; LICA: left internal carotid artery; LSTA: left superior thyroid artery; CD: chow diet; HFD: high fat diet; Lig.: ligation.

Sex-matched groups weighing 20–25g were enrolled in this study. Male apolipoprotein E deficient mice (*Apoe* ^*-/-*^) (8 weeks-old) were subjected to combined partial ligation of the left common carotid artery (LCCA) and the left renal artery (LRA, n = 10, Fig 1A) or cast placement around the LCCA and partial ligation of the LRA (n = 10, Fig 1B). Females (8 weeks-old) were either subjected to partial ligation of the LCCA and cast placement (n = 10, Fig 1C) or only cast implantation (n = 10, Fig 1D). Mice were fed *ad libitum* with chow diet (CD) or high fat diet (HFD, 22% fat and 0.15% cholesterol, abdiets, Weerden, The Netherlands) when indicated and during the periods described in Fig 1.

### Surgery procedure

A detailed listing of the methodological modifications from the originally reported intervention is provided in the online supplemental materials (Tables A-D in S1 File). All mice undergoing surgery were injected s.c. with Temgesic 30min before surgery and in the evening after to alleviate the suffering during the surgical procedure and to ensure the post-surgical recovery.

#### Partial ligation of the left common carotid artery

The partial ligation of the LCCA was performed as described by [10]. In brief, mice were anesthetized with isoflurane via inhalation through a nose mask and were maintained throughout the surgery at 37°C on a heating pad. Next, the anterior cervical triangles were accessed by a sagittal anterior neck incision. The LCCA was individualized from circumferential connective tissues by blunt dissection and the exposed branches of the LCCA but not the superior thyroid artery (LSTA) was permanently ligated with a surgical suture (7–0 silk) (Fig 1A and 1C). After validating that blood flow was present through the LCCA the incision was closed with suture (6–0 silk).

#### Partial ligation of the left renal artery

Mice were subjected to endogenous renovascular hypertension using modified LRA ligation as described by Jin et al. [8]. In brief, mice were anesthetized with isoflurane via inhalation through a nose mask and were maintained throughout the surgery at 37°C on a heating pad. After a small flank incision the left kidney was exposed and the LRA was ligated with a surgical suture (6–0 silk) along with a spacer (outer diameter 0.11mm) (Fig 1A and 1B). Subsequently, the spacer was removed leaving a tight stenosis in the LRA. Thereafter, the kidney was gently placed back into the retroperitoneal cavity and the muscle layer and the skin were closed with suture (6–0 silk). To test the successful induction of endogenous renovascular hypertension, significant shrinkage of the left kidney was observed (Figure A in S1 File). In addition, systolic blood pressure was measured using a sphygmomanometer and the tail-cuff method before the surgery and every 2 weeks after the surgery (Figure A in S1B–S1E File).

#### Cast placement around the left common carotid artery

To induce local changes in shear stress in the LCCA we used the cast as described by Cheng and colleagues [9]. This device consists of two longitudinal halves of a cylinder with a cone-shaped lumen (constructive diameter 0.2mm) (Fig 1B–1D). Mice were anesthetized with isoflurane via inhalation through a nose mask and were maintained throughout the surgery at 37°C on a heating pad. The anterior cervical triangles were accessed by a sagittal anterior neck incision. The LCCA was individualized from circumferential connective tissues by blunt dissection and the cast was placed around the LCCA. After validating that blood flow was present through the LCCA the incision was closed with suture (6–0 silk).

### Tissue processing

Mice were euthanized by CO_2_ inhalation in a euthanasia chamber. After collecting blood via heart puncture, animals were perfused with 20ml PBS, pH 7.4. Next, the LCCA was removed and embedded in OCT compound (Tissue-Tek, Sakura Finetek, Torrance, CA). Samples were frozen on isopentane and liquid nitrogen and stored at -20°C. Haematologic counts were determined with the ScilVet abc Plus analyzer (scil animal care company GmbH, Viernheim, Germany) (Figure B in S1 File).

### Histological and Immunofluorescence staining

Cryosections (7μm) were histologically stained with standard hematoxylin and eosin (H&E) in intervals of 70μm. Consecutive sections were stained with picrosirius red for collagen and Pearls’ Prussian blue staining for identification of intraplaque hemorrhages (IPH). Collagen fiber composition was evaluated using polarized light illumination. MMP9 immunohistochemistry was performed using a ´mouse-on-mouse´ strategy as described in Goodpaster *et al* [11]. In brief, cryosections were air-dried and fixed with cold acetone. Mouse anti-human MMP9 (R&D, Minneapolis, USA) was pre-incubated with a rabbit Fab anti-mouse IgG (Rockland, Limerick, USA) antibody and added to the cryosections in a dilution (1:100). After extensive washing, sections were incubated with BrightVision poly-horseradish peroxidase-anti-rabbit IgG (Immunologic BV, Duiven, The Netherlads) and visualized by DAB (3,3' diaminobenzidine, ImmunoLogic, Duiven, The Netherlands). Counterstaining for the nuclei was performed by incubation with hematoxylin. For immunofluorescence staining, cryosections were air-dried and fixed with cold acetone. Next, specimens were blocked with 5% horse serum/PBS and incubated with primary antibodies rabbit anti-mouse CD68 (1:200, Abcam, Cambridge, MA), rat anti-mouse CD86 (1:500, BioLegend, San Diego, USA), alexa Fluor 647-conjugated rat anti-mouse CD301 (1:500, AbD Serotec, Kidlington, UK) and FITC-conjugated mouse anti-mouse smooth muscle cell α-actin (SMA, 1:1000, Sigma-Aldrich, St. Louis, MO). After extensive washing, sections were incubated with Alexa Fluor 488 or 647 donkey anti-rabbit and Alexa Fluor 594 donkey anti-rat (1:500, Invitrogen, Breda, The Netherlands). Counterstaining for the nuclei was performed by incubating with DAPI (Invitrogen). Images were acquired with a laser microscope (TCS/SP8, Leica, Wetzlar, Germany).

### Atherosclerotic plaque analysis

Atherosclerotic plaque analysis was performed on sections recollected after the suture in the LCCA LRA and LCCA Cast models and in the low shear stress region generated by cast placement in LRA Cast and Cast models. Carotid samples showing thrombus or absence of lesion formation were excluded from this analysis. Histological sections were quantified by computer-assisted morphometric analysis using ImageJ software (National Institutes of Health, Bethesda, MD). Intima to media ratio was calculated as the quotient of intima and media areas (μm^2^). Necrotic cores (NC) were defined as area absent of nuclei underneath a formed fibrous cap (FC). Since tissue loss was observed in H&E compared to picrosirius red stained sections, the NC core was assessed using these different staining (Figure C in S1 File). FC thickness was defined as the average of lengths measurements in the positions overlapping with the lines of a square-shaped grid.

### Lesion classification

Lesions were scored blinded by two independent, very experienced pathologists with little inter- and intra-variability. The classification was based on the following scoring: early, accumulation of VSMCs, absence of lipid or macrophage foam cells; advanced type I, multiple layers of macrophage foam cells; advanced type II, multiple layers of macrophage foam cells, well-formed NC covered by a thin FC, rare VSMCs and collagen content, eventually IPH; advanced type III, neointimal thickening, highly increased intimal thickening, high amount of VSMCs and macrophages, partial NC formation; complicated, presence of luminal thrombus (Table E in S1 File).

### Vulnerability-Index (VI and VI_c_)

Vulnerability-Index (VI) was used to evaluate the overall degree of vulnerability between the models by integrating all plaque features analyzed in the study. VI was adapted from [12]. VI was calculated by the relation between analyzed unstable (*U*) and stable (*S*) features of the plaque and corrected by the incidence of lesion formation (p, VI_c_). The formula for VI was expressed as *VI*_*c(i)*_ = *(U*_*(i)*_ */S*_*(i)*_ *)*, where *i* represents each studied mouse. The formula for corrected VI was expressed as *VI*_*c(i)*_ = *(U*_*(i)*_ */S*_*(i)*_ )*p_(i),_ where *i* represents each studied mouse_._ U includes the sum of NC area (% of intima), CD68+ area (% of intima) and of Pearls’ Prussian blue+ area (% of intima). S consists of SMA+ area (% of intima) and collagen+ area (% of intima). Analysis of VI and VI_c_ were performed on lesions with the same severity score of type II (see above). Lesions not representing the type II phenotype were scored as zero.

### Statistical analysis

All data were represented as individual data points along with mean values. For the individual parameters was no statistical analysis performed due to the evaluation of the features of the regarded model itself. Statistical analysis was performed for the VI_c_ as comparison of the models among themselves. Statistical analysis was done with GraphPad Prism 5 (GraphPad Software, LaJolla, CA). Unpaired Student’s *t*-test or Mann-Whitney (one variable) or one-way ANOVA with Bonferroni’s Multiple Comparison test (> 2 variables) were applied, as appropriate. p-values < 0.05 were considered as being statistically significant.

## Results

### Mouse model description and incidence of lesion formation

**Fig 2:**
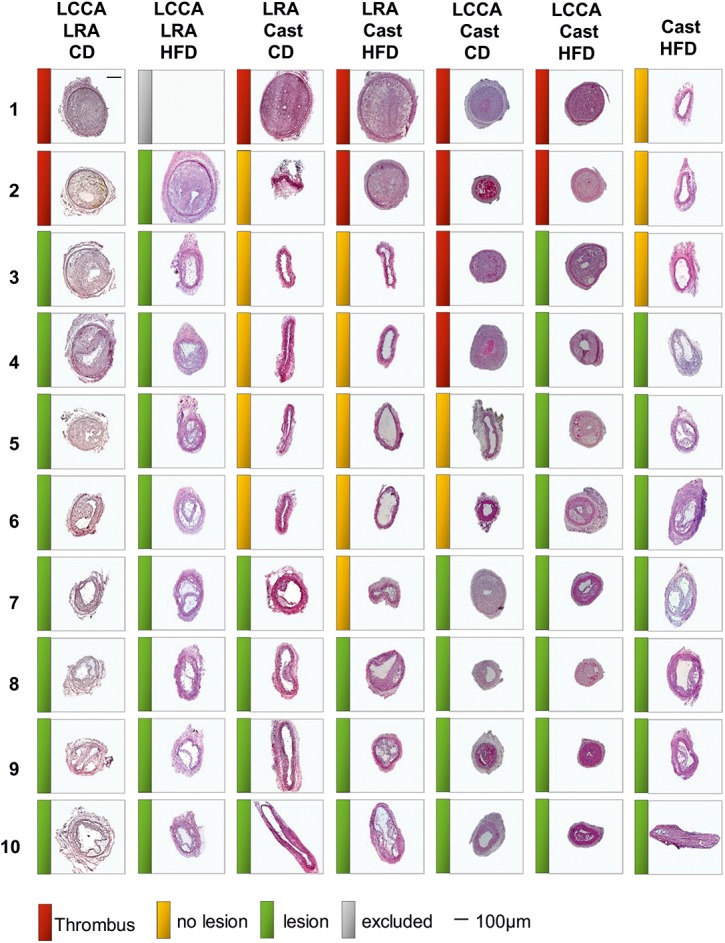
Representative carotid cross-sections of studied specimens for the mouse models of atherosclerotic plaque destabilization. Mice were fed chow diet (CD) or high fat diet (HFD) as indicated. From left to right: LCCA LRA, model based on the combination of partial ligation of LCCA and LRA; LRA Cast, model based on the combined cast placement around LCCA and partial ligation of LRA; LCCA Cast, model based on the partial ligation of LCCA in combination with the cast placement around the LCCA; Cast, model based on cast placement around the LCCA. Red, yellow and green bars represent thrombus, no lesion or atherosclerotic lesion, respectively. Scale bar = 100μm. LCCA: left common carotid artery; LRA: left renal artery; CD: chow diet; HFD: high fat diet.

We performed 4 different mouse models of atherosclerotic plaque destabilization adapted from previous described studies [7–9]. First, *Apoe* ^*-/-*^ mice were subjected to alteration of blood flow in the LCCA by partial ligation (Fig 1A) or implantation of a shear stress modifier (cast, Fig 1B) as described in [9]. Hypertension has been shown to increase signs of plaque vulnerability, thus we performed partial ligation of the LRA in combination with mentioned blood flow alterations [8]. We observed successful induction of hypertension as determined by a significant increase in the systolic blood pressure 2 weeks after the surgery and the shrinkage of the left kidney (Figure A in S1 File). In addition, we performed another model based on alteration of the blood flow by partial ligation and subsequent implantation of a cast in the LCCA of *Apoe* ^*-/-*^ mice (Fig 1C). This method generates a diminished blood flow in the carotid bifurcation and the low shear-stress region of the cast accelerating the formation of the atherosclerotic lesion, as described in [7]. Of note, we substituted the cuff for the cast implantation in order to generate the lesion in a more predictable location as observed with the fixed constructive diameter (0.2mm) of the cast [7]. All these models were performed under CD or HFD. Finally, we studied a model solely based on cast implantation in the LCCA of *Apoe* ^*-/-*^ mice under HFD regime as described by Cheng et al. (Fig 1D). As shown in Fig 2, we observed a difference in the incidence of atherosclerotic plaque formation (including VSMC rich neointimas) between the different models: LCCA LRA (CD: 80%, HFD: 89%), LRA Cast (CD: 40%, HFD: 30%), LCCA Cast (CD: 40%, HFD: 80%) and Cast HFD: 70%. Absence of atherosclerotic plaque was defined as the presence of a non-lesion associated luminal thrombus or the absence of lesions (non-responders) in the left carotid artery.

### Analysis of structural features of the atherosclerotic plaques

**Fig 3:**
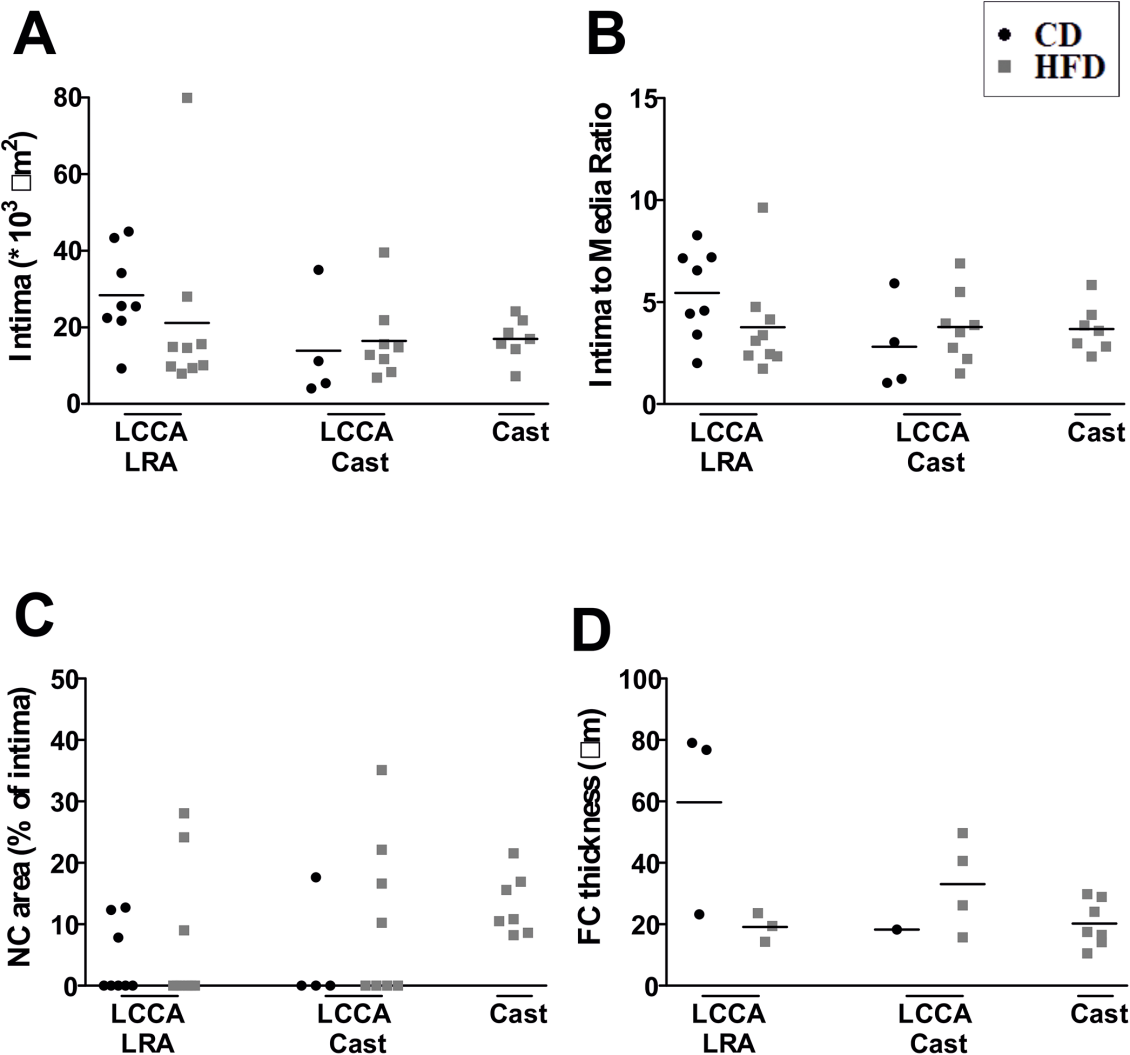
Structural features of the atherosclerotic lesions. Quantification of (**A**) intimal area, (**B**) intima to media ratio, (**C**) necrotic core size and (**D**) fibrous cap thickness. Fibrous cap thickness was only measured when necrotic core was present. NC: necrotic core; FC: fibrous cap; CD: chow diet; HFD: high fat diet; LCCA: left common coronary artery; LRA: left renal artery.

Additionally to the incidence of lesion formation, we examined the structural phenotype of formed lesions. Of note, carotid samples showing thrombus or absence of lesion formation were excluded from this analysis. Based on this criteria, the number of excluded carotid samples in the LRA Cast model (CD and HFD) comprised the majority of studied samples, therefore the following analysis were not performed in these models. First, we analyzed lesion size defined as intima to media ratio. Assessed mouse models exhibited differential intima to media ratios: LCCA LRA (CD: 5.5 ± 2.2, HFD: 3.8 ± 2.4), LCCA Cast (CD: 2.8 ± 2.2, HFD: 3.8 ± 1.8) and Cast HFD: 3.7 ± 1.2 (Fig 3B). Besides an expanded plaque size, enlarged NC and thin FC better define human vulnerable plaques [3]. Thus, we explored the NC formation in sections of studied specimens. Overall, we found different incidence of NC formation (0–70%) and variations in size (%NC of intimal area): LCCA LRA (CD: 10.9 ± 2.8%, HFD: 20.4 ± 10.0%), LCCA Cast (CD: 17.7%, HFD: 21.0 ± 10.6%) and Cast HFD: 13.2 ± 5.0% (Fig 3C). In the sections where a NC was present, FC thickness was measured showing: LCCA LRA (CD: 59.7 ± 31.6μm, HFD: 19.1 ± 4.7μm), LCCA Cast (CD: 18.3μm, HFD: 33.1 ± 15.1μm) and Cast HFD: 20.2 ± 7.4μm (Fig 3D).

### Analysis of compositional features of the atherosclerotic plaques

**Fig 4:**
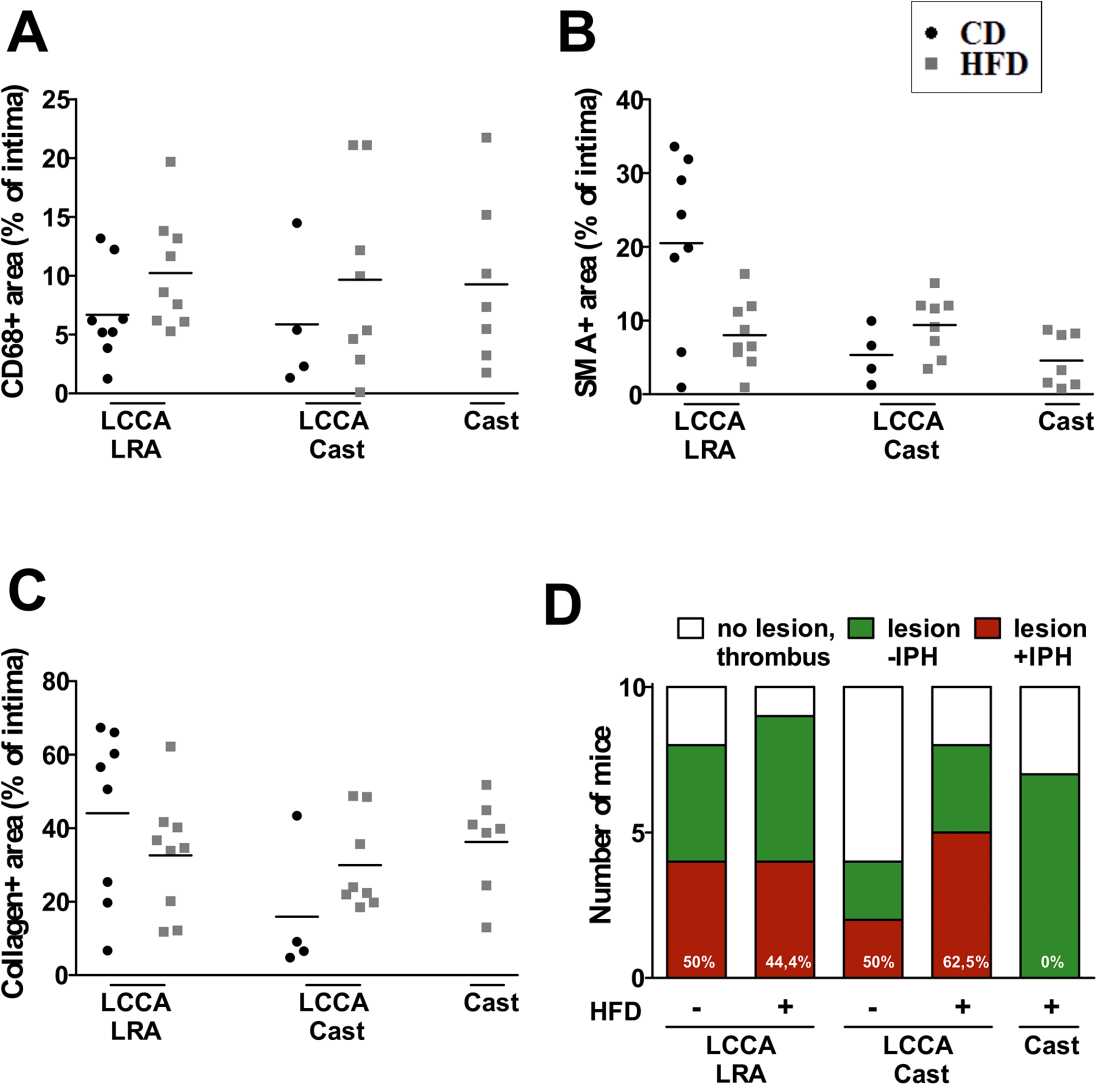
Compositional features of the atherosclerotic lesions. Quantification of (**A**) macrophage (CD68+ area), (**B**) vascular smooth muscle cell (SMA+ area) and (**C**) total collagen (picrosirius red+ area) content. (**D**) Quantification of the incidence of intraplaque hemorrhage in Pearls’ Prussian blue stained sections. CD: chow diet; HFD: high fat diet; LCCA: left common coronary artery; LRA: left renal artery; IPH: intraplaque hemorrhages.

Increased macrophage infiltration followed by decrease in VSMC and collagen content are important indicators of plaque vulnerability [4]. Macrophage content detected by CD68 immunostaining revealed variable infiltration into the intima: LCCA LRA (CD: 6.7 ± 4.0%, HFD: 10.2 ± 4.8%), LCCA Cast (CD: 5.9 ± 6.0%, HFD: 9.7 ± 8.0%) and Cast HFD: 9.3 ± 6.0% (Fig 4A). Additionally, the phenotype of the macrophages was characterized by analyzing CD86 (marker of M1 or classical phenotype) and CD301 (marker of M2 or alternative phenotype) for the models LCCA LRA CD and Cast HFD (Figure D in S1 File). Analysis of the CD68 and CD86 positive macrophage content showed an increase in Cast HFD compared to LCCA LRA CD (5.47 ± 2.55% vs 10.98 ± 2.39%). On the other hand, a decrease in CD68 and CD301 positive macrophages was detected in the model Cast HFD compared to LCCA LRA CD (0.55 ± 0.23% vs 0.11 ± 0.04%) (Figure D in S1 File). Furthermore, we analyzed lesional VSMC content, an indicator of plaque stability. Quantification of intimal SMA area resulted as follows: LCCA LRA (CD: 20.5 ± 12.0%, HFD: 8.0 ± 4.6%), LCCA Cast (CD: 5.3 ± 3.8%, HFD: 9.4 ± 4.0%) and Cast HFD: 4.6 ± 3.6% (Fig 4B). Next, we measured intimal collagen by picrosirius red staining. The percentage of collagen positive area within the intima was: LCCA LRA (CD: 44.1 ± 23.4%, HFD: 35.6 ± 16.0%), LCCA Cast (CD: 15.9 ± 18.4%, HFD: 29.9 ± 12.6%) and Cast HFD: 36.2 ± 13.2% (Fig 4C). Detailed analysis of the collagen content demonstrates a significant difference between thin and thick fibers for the model Cast HFD (thick fibers: 71.92 ± 7.47%, thin fibers: 24.60 ± 6.75%), whereas no significant difference was shown for the model LCCA LRA CD (thick fibers: 42.64 ± 8.23%, thin fibers: 54.39 ± 8.32%) (Figure E in S1 File). Additionally, it has been demonstrated that matrix metalloproteinases (MMPs) degrade a broad range of matrix substrates like elastin, collagen and fibronectin [13]. Thus we analyzed the content of MMP9 within the lesions of the models LCCA LRA CD and Cast HFD (Figure F in S1 File). A significant content increase of MMP9 was detected in the model Cast HFD compared to LCCA LRA CD (10.98 ± 3.26% vs 31.75 ± 8.45%) (Figure F in S1 File). Neoangiogenesis and IPH are independent factors associated with plaque instability and thrombotic events [14]. Detection of IPH has been previously described in different mouse models of atherosclerotic plaque destabilization [8, 9, 15]. Hence, we aimed to assess the presence of IPH in our mouse models. IPHs were frequently occurring in the models LCCA Cast (CD: 50%, HFD 62.5%) and LCCA LRA (CD: 50%) (Fig 4D), whereas in the model Cast HFD IPH was undetectable. On the other hand, analysis of neovessels by CD31 staining was negative in all samples analyzed (data not shown). This discrepancy has been also described by others [16].

### Plaque classification and determination of the Vulnerability-Index

To integrate all parameters analyzed, we first classified the atherosclerotic lesions as described in Material and Methods as our type II lesions that represents vulnerable plaques with thin fibrous cap atheroma (TCFA) phenotype (Table E in S1 File). Except for LRA Cast under chow diet, all models exhibited type II advanced plaques (Figure G in S1 File). We next calculated the vulnerability-index that allowed us to integrate all parameters analyzed and determine the degree of vulnerability between the models. The index includes the components of increasing and decreasing factors of plaque instability (Table 1). After correction with the incidence of the type II lesion formation (see Material and Methods), we calculated the following indices: LCCA LRA (CD: 0.004 ± 0.01, HFD: 0.07 ± 0.1), LCCA Cast (CD: 0.02 ± 0.03, HFD: 0.12 ± 0.18) and Cast HFD: 0.45 ± 0.36 (Table 1).

**Table 1:** Vulnerability-Index of the lesions in the mouse models of atherosclerotic plaque destabilization.

	Vulnerability-Index (VI)	Incidence of type II	Corrected Vulnerability-Index (VI_c_)	p-value (vs Cast HFD)
LCCA LRA CD	0.32	1/10	0.004 ± 0.01	< 0.001
LCCA LRA HFD	0.55±0.32	3/9	0.07 ± 0.1	< 0.01
LCCA Cast CD	0.61	1/10	0.02 ± 0.03	< 0.01
LCCA Cast HFD	1.05±0.54	3/10	0.12±0.18	< 0.05
Cast HFD	0.65±0.51	7/10	0.45±0.36	-

Vulnerability-Index (VI) was calculated by the relation between analyzed unstable (*U*) and stable (*S*) features of the plaque and corrected by the incidence of lesion formation (p, VI_c_). Lesions not representing the type II phenotype were scored as zero. SD was not indicated for models with incidence of n = 1 of type II lesions. LCCA: left common coronary artery; LRA: left renal artery; CD: chow diet; HFD: high fat diet. (n = 3–8; p < 0.05, p < 0.01, p < 0.001 with 1-way ANOVA with Bonferroni’s Multiple Comparison test)

## Discussion

Rupture of human culprit atherosclerotic plaques is preceded by the conversion of a stable into an unstable atherosclerotic plaque. Post-mortem analyses of human specimens have defined the characteristics of the archetypical vulnerable plaques responsible for most of the thrombotic events associated with ACS. However, the study of the mechanisms underlying the process of atherosclerotic plaque destabilization has been hampered by the lack of proper animal models. Reported mouse models of atherosclerotic plaque destabilization describe the formation of advanced atheromas exhibiting different signs of plaque instability such as NC expansion, FC thinning and an inflammatory phenotype [4]. Additional destabilizing features such as neoangiogenesis or IPH [7, 9, 15] are eventually detected while others such as plaque disruption with superimposed thrombosis can hardly be identified [15, 17, 18]. Indeed, it is well accepted that mouse atherosclerotic plaques only partly resemble the characteristics observed in human advanced lesions. However, the advantages of identifying novel mechanistic pathways in experimental atherosclerosis models highlight the importance of establishing a consensus model of plaque destabilization in mice.

The goal of the present study was to compare different mouse models of atherosclerotic plaque destabilization to analyze the degree of lesion instability and the robustness of the model. Elevated blood pressure is a major risk of cardiovascular diseases and blood-pressure lowering therapies significantly reduce events of ACS [19]. Induction of hypertension by angiotensin II infusion or constriction of the renal artery has been shown to increase signs of vulnerability in mouse models of advanced atherosclerosis [8, 9]. Hence, we performed a mouse model based on renovascular hypertension by partial ligation of the LRA combined with induction of low shear stress in the carotid artery through partial ligation of or implantation of a shear stress modifier (cast) in the LCCA. Under chow diet regime, partial ligation of the LRA and LCCA generated neointimal thickening-type atherosclerotic lesions with expanded size and VSMC-enriched phenotype compared to HFD feeding condition. In line with these results, angiotensin II infusion has been shown to increase VSMC response by increasing proliferation and migration leading to neointimal thickening [20]. The formation of a NC, thinning of the FC, increased macrophage infiltration and the reduction of collagen and VSMC content, which are critical factors of human plaque destabilization [3], were detected in a lower extent. Overall, based on our criteria the features that define human-like plaque vulnerability are not fulfilled by the LCCA LRA model under CD. On the other hand, these factors were induced by HFD feeding, probably through increasing macrophage infiltration, leading to a more vulnerable phenotype. Under hypertensive conditions, alteration of the blood flow by implantation of a cast in the LCCA exhibited a reduced incidence of lesion formation compared to partial ligation of the LCCA both under CD or HFD regime. Intense reduction of the blood flow by partial ligation of the LCCA compared to that generated by the constrictive diameter of the cast (0.2 mm) may explain the differences in the incidence of lesion formation. Moreover, out of all identified lesions, a predominance of early neointimal thickening lesions or type I advanced lesions was observed, suggesting lower progression of the atherosclerotic disease. A limitation of the study is that although observing a significant increase of the blood pressure 2–4 weeks after LRA partial ligation, the blood pressure in models LCCA LRA and LRA Cast under HFD was subsequently stabilized. Hence, the time-point of analysis may be suboptimal since the effect of hypertension on atherosclerotic plaque destabilization was no longer present.

In addition, we evaluated a mouse model of atherosclerotic plaque destabilization involving the partial ligation of LCCA followed by the implantation of a constrictive cast four days before sacrifice. Compared to the model previously reported by Sasaki and colleagues [7], we substituted the use of a polyethylene cuff for a cast to reduce the variability in the extent of artery constriction. As reported by Sasaki et al [7] and as observed in the model involving partial ligation of the LCCA and LRA, mice developed VSMC-enriched neointimal thickening lesions under CD feeding. Furthermore, in agreement with Sasaki’s work, we evidenced a higher presence of occlusive or mural thrombus. As mentioned above, human plaque rupture requires the contact of NC content with the vessel lumen through a disrupted FC which can be associated with the formation of a superimposed thrombus. Based on this definition, we however could not identify signs of plaque disruption as a cause of the observed thrombus formation. Of note, the use of a constrictive cast instead of the polyethylene cuff may explain these observations. Similarly, thrombus formation in the model involving partial ligation of the LCCA and LRA was neither associated to plaque disruption. As previously observed feeding with HFD boosted inflammatory macrophage infiltration, increased NC formation and reduced the number of excluded specimens due to thrombus formation or absence of lesion formation.

Finally, we used the model described by Cheng and colleagues where *Apoe* ^*-/-*^ mice were subjected to the implantation of a cast in the LCCA [9]. As predicted, advanced atherosclerotic lesions where formed in the cast region with low shear stress in respect to the high shear or oscillatory stress regions (data not shown). This model involved a longer period of HFD feeding (11 weeks) which was associated with the formation of TFCA with expanded NC and thinner FC and exhibiting an inflammatory phenotype.

Adequate assessment of atherosclerotic plaque vulnerability enables us to integrate data obtained from the study of different structural and compositional factors with functional implication in plaque instability. We have developed an index of plaque instability (VI) to quantitatively study lesion severity and vulnerability in a simple way. This index includes a ratio of destabilizing factors such as NC size, macrophage content or IPH presence and factors with a stabilizing function like VSMC and collagen content. This ratio was combined with the incidence of the vulnerable type II lesions in the respectable model. Of note, other criteria could be included in this index that can be relevant in different experimental setups. In order to reduce statistical variability and obtain more reliable results, we first classified the lesions according to their severity degree using elements of the Virmani histopathological classification. For instance, the partial ligation of the LCCA in combination with renovascular hypertension model was characterized by a notable variability in the severity degree between the different studied specimens. Moreover, different lesion types were found within the carotid artery surface (data not shown) thereby increasing the difficulty to define the individual degree of vulnerability and augmenting the variability for each parameter analyzed. Since, advanced atheroma type II (TFCA) resemble a higher number of characteristics associated with human atherosclerotic vulnerable plaques we focused our analysis on this lesion type. Models based on partial ligation of the LCCA combined with cast implantation under HFD regime or solely cast placement showed a higher index of plaque instability. However, although exhibiting an increased index of plaque instability, type II advanced plaques were found in lower extent in the model LCCA Cast on HFD compared to the cast model. After correction of the index [3] with the incidence of vulnerable plaque formation, the Cast model reflected a higher overall vulnerability in compared to LCCA Cast on HFD model.

Collectively, we have observed that the mouse model based on the cast implantation under hypercholesterolemia exhibited increased incidence of human-like atherosclerotic vulnerable plaques with less variability between the analyzed specimens (Table 2). It is worth mentioning, that all models displayed different instability traits associated with human atherosclerotic plaque vulnerability. For instance, we were not able to detect IPH in the cast HFD model compared to the other models, although Cheng et al. reported a spontaneous or angiotentin II-induced incidence of 29% vs 75%. This variation may be due to different staining and analysis techniques [9]. In addition, we believe that the different gender has no impact on the development of the lesions type II, based on the study of Jin et al. using sex-matched groups [8]. Taken together these findings highlight the fact that the selection of a model will depend of the research question that is going to be addressed (Table 2). For example, the duration of the model may limit the application of drug/antibody treatments that can only be applied for a specific maximum period of time. Furthermore, mice undergoing two sequential surgeries as LRA/LCCA followed by cast placement may display a higher distress which could have an additional impact on the results (Table 2). Of note, the models analyzed in this study did not exhibit signs of thrombotic events associated either with plaque disruption or erosion. This aspect highlights the previously described limitations of mouse models in studying thrombotic events and subsequent clinical outputs such as myocardial infarction or stroke [16]. Nonetheless, events occurring during human atherothrombosis (late stages of atherosclerotic disease) preceding plaque rupture, such as NC formation, lesional cell-death or fibrotic tissue degradation are still detected in these mouse models. An additional possibility to study plaque vulnerability would be the use of humanized mouse. These humanized mouse models have been shown to possess different capabilities of engrafting human hematolymphoid cells [21, 22]. But they bring also limitations, thus it would not include the impact of SMC or collagen content in the progression of the plaque destabilization and could just give information about the impact of hematolymphoid cells. Interestingly, the vast use of mouse models to study atherogenesis has resulted in tremendous progress in the identification of novel molecular targets for treatment of early atherosclerosis. However its clinical application has been restricted since patients are not usually treated during early phases of the disease. On the other hand, mouse models of atherosclerotic plaque destabilization may help to identify molecular mechanisms involved in advanced atherosclerosis development that could be more easily translated to the clinical practice. Combination of this knowledge with novel imaging techniques and biomarker screening may allow for earlier detection and subsequent treatment of patients with subclinical silent vulnerable lesions before resulting in fatal ACS.

**Table 2:** Summary of features of the plaque destabilization models.

	LCCA LRA CD	LCCA LRA HFD	LRA Cast CD	LRA Cast HFD	LCCA Cast CD	LCCA Cast HFD	Cast HFD
Technical challenge	•••	•••	••	••	••	••	•
Duration	••	••	••	••	•	•	•••
Non-Responders	••	•	•••	•••	•••	••	••
Robustness (VI, VI_c_)	•	••	-	-	•	•••	•••
Human-like	•	•	-	-	•	••	•••
Incidence of							
-mural Thrombus	••	-	-	-	•••	••	-
-Lesion	•••	•••	-	-	•	•••	•••
-TFC/NC	••	••	-	-	•	••	•••
-IPH	•••	•••	-	-	•••	•••	-

• low •• medium ••• high—n/a

LCCA: left common coronary artery; LRA: left renal artery; CD: chow diet; HFD: high fat diet; VI: Vulnerability-Index; VI_c_: corrected Vulnerability-Index; FC: fibrous cap; NC: necrotic core; IPH: intraplaque hemorrhage.

## Supporting Information

S1 ARRIVE Checklist ARRIVE Guidelines Checklist. (DOC)

S1 File Supporting information Hartwig et al. (DOC)
