# Supplementary material for: Atherosclerotic Plaque Destabilization in Mice: A Comparative Study
Source: PLoS One. 2015 Oct 22;10(10):e0141019. doi: 10.1371/journal.pone.0141019 (PMC4619621; doi:10.1371/journal.pone.0141019)
Supplement: S1 ARRIVE Checklist — (DOC) [file pone.0141019.s001.doc]

**S13: ARRIVE – Animal Research Reporting in vivo experiments**

**1. Title**

Atherosclerotic plaque destabilization in mice: A comparative study

**2. Abstract**

**Background**

Thrombotic events associated to rupture of vulnerable atherosclerotic plaques are responsible of the majority of acute coronary events, main cause of mortality and morbidity in developed countries. Rupture-prone plaques are characterized by the presence of an expanded necrotic core, covered by a thin fibrous cap and exacerbated inflammation. This knowledge derives from post-mortem analysis of human specimens. However, the understanding of the mechanisms underlying the conversion of a stable into an unstable plaque is scarce. Hence, the development of proper mouse models mimicking the processes occurring during atherosclerotic plaque destabilization may be helpful in identifying novel targets with potential application in detection and treatment of acute coronary syndromes.

**Experimental approach**

Male ApoE^-/-^ mice (8 weeks-old) were subjected to combined partial ligation of the left common carotid artery (LCCA) and the left renal artery (LRA) or cast deployment around the LCCA and partial ligation of the LRA. Females (8 weeks-old) were either subjected to partial ligation of the LCCA and cast deployment or only cast implantation. Mice were fed *ad libitum* with chow diet (CD) or high fat diet (HFD, 22% fat and 0.15% cholesterol, abdiets, Weerden, The Netherlands) during the periods of 6-11 weeks regarding the model.

**Key findings**

Alteration of the local shear stress was induced in apolipoprotein E deficient mice by partial ligation of and/or the implantation of a shear stress modifier device (cast) in the left common carotid artery. In addition, endogenous renovascular hypertension was induced through partial ligation of the left common carotid artery. Moreover, we examined the effect of hypercholesterolemia on the plaque progression by feeding the mice chow or high fat diet. Overall analysis of the incidence of vulnerable plaque formation and their structural and compositional traits suggested that models based on partial ligation of the left common carotid artery in combination with the cast placement or solely cast placement, both under high fat diet regime display increased vulnerability degree and reproducibility.

**Conclusions and Implications**

Comparative analysis of the atherosclerotic plaque phenotype and reproducibility of different mouse models of the atherosclerotic plaque destabilization may help researchers to select the most suitable model to address an specific research question. Taking advantage of these mouse models may help to identify novel molecular mechanisms involved in advanced atherosclerosis development that could be translated to the clinical practice. Combination of this knowledge with novel imaging techniques and biomarker screening may allow for earlier detection and subsequent treatment of patients with subclinical silent vulnerable lesions before resulting in fatal acute coronary syndromes.

**3. INTRODUCTION**

**a. Background**

Atherosclerosis is a chronic inflammatory response to modified lipids in the arterial vessel wall and the main cause of morbidity and mortality in western societies. It is a dynamic progression from early to rupture-prone plaques, which are characterized by exacerbated infiltration of inflammatory cells combined with a large necrotic core covered by a thin fibrous cap (1). Structural damage in the FC results in the exposure of the highly thrombogenic material of the necrotic core to the blood, leading to thrombus formation, occlusion of the coronary artery, and associated acute coronary syndromes (2). Albeit the clinical relevance of this process, the understanding of the mechanisms underlying the conversion of a stable into an unstable and ruptured plaque remains unclear. Various mouse models based in surgical approaches have been proposed (2). Although these models generate atherosclerotic plaques with histological features of human advanced lesions, a consensus model to study atherosclerotic plaque destabilization is still lacking (3). Therefore we employed two methods of local shear stress alteration based on the partial ligation of and/or the implantation of a shear stress modifier device in the left common carotid artery. In addition, we induced endogenous renovascular hypertension through partial ligation of the left renal artery. Finally, we examined the effect of aggravated hypercholesterolemia on plaque development in these models by feeding the mice chow or high fat diet.

**b.**

As a murine model we utilized *ApoE-/-* mice. These have a defect in their lipid-metabolism, which contributes to the development of atherosclerotic lesions, comparable to the human. In addition, to facilitate atherosclerotic lesion formation, *ApoE-/-* mice received a High Fat Diet (HFD; 22% fat and 0.15% cholesterol) (4).

**4. Objectives**

We aimed to compare different surgical approaches of plaque destabilization and to evaluate the degree of plaque vulnerability in these mouse models and finally to evaluate their reproducibility.

**METHODS**

**5. Ethical Statement**

This study was carried out in strict accordance with the recommendations in the Guide for the Care and Use of Laboratory Animals of the National Institutes of Health. The protocol was approved by the Committee on the Ethics of Animal Experiments of the University of Amsterdam and the Animal Research Institute (ARIA), Amsterdam (Permit Number: DBC102939 and DBC102940). All surgeries were performed under isoflurane anesthesia, and all efforts were made to minimize suffering.

**6. Study design**

Male *Apoe^-/-^* were subjected to combined partial ligation of the left common carotid artery (LCCA) and the left renal artery (LRA, n=10, Fig. 1A) or cast deployment around the LCCA and partial ligation of the LRA (n=10, Fig. 1B). Females were either subjected to partial ligation of the LCCA and cast deployment (n=10, Fig. 1C) or only cast implantation (n=10, Fig. 1D). Mice were fed *ad libitum* with chow diet (CD) or HFD when indicated and during the periods described in Figure 1A-D.

Sections were scored blinded by two independent, very experienced pathologists with little inter- and intra-variability

**Figure 1. Experimental setups of the mouse models of atherosclerotic plaque destabilization**. (**A**) Model based on the combined partial ligation of LCCA and LRA. (**B**) Model based on the combination of cast deployment around LCCA and partial ligation of LRA. (**C**) Model based on the partial ligation of LCCA in combination with the cast placement around the LCCA. (**D**) Model based on cast deployment around the LCCA. LCCA: left common carotid artery; LRA: left renal artery; LECA: left external carotid artery; LICA: left internal carotid artery; LSTA: left superior thyroid artery; CD: chow diet; HFD: high fat diet; Lig.: ligation.

**7. Experimental procedure**

*Partial ligation of the left common carotid artery*

The partial ligation of the LCCA was performed as described by (5). In brief, mice were anesthetized with isoflurane via inhalation through a nose mask and were maintained throughout the surgery at 37°C on a heating pad. Next, the anterior cervical triangles were accessed by a sagittal anterior neck incision. The LCCA was individualized from circumferential connective tissues by blunt dissection and the exposed branches of the LCCA but not the superior thyroid artery (LSTA) was permanently ligated with a surgical suture (7-0 silk). After validating that blood flow was present through the LCCA the incision was closed with suture (6-0 silk).

*Partial ligation of the left renal artery*

Mice were subjected to endogenous renovascular hypertension using modified LRA ligation as described by Jin et al. (6). In brief, mice were anesthetized with isoflurane via inhalation through a nose mask and were maintained throughout the surgery at 37°C on a heating pad. After a small flank incision the left kidney was exposed and the LRA was ligated with a surgical suture (6-0 silk) along with a spacer (outer diameter 0.11mm. Subsequently, the spacer was removed leaving a tight stenosis in the LRA. Thereafter, the kidney was gently placed back into the retroperitoneal cavity and the muscle layer and the skin were closed with suture (6-0 silk). To test the successful induction of endogenous renovascular hypertension, significant shrinkage of the left kidney was observed. In addition, systolic blood pressure was measured using a sphygmomanometer and the tail-cuff method before the surgery and every 2 weeks after the surgery.

*Cast deployment around the left common carotid artery*

To induce local changes in shear stress in the LCCA we used the cast as described by Cheng and colleagues (7). This device consists of two longitudinal halves of a cylinder with a cone-shaped lumen (constructive diameter 0.2mm). Mice were anesthetized with isoflurane via inhalation through a nose mask and were maintained throughout the surgery at 37°C on a heating pad. The anterior cervical triangles were accessed by a sagittal anterior neck incision. The LCCA was individualized from circumferential connective tissues by blunt dissection and the cast was placed around the LCCA. After validating that blood flow was present through the LCCA the incision was closed with suture (6-0 silk).

**8. Experimental animals**

Male and female *Apoe^-/-^* mice (8 weeks old; n=10 per group) from own breeding were included.

**9. Housing and Husbandry**

Animals were housed with an inverse 12 hours day-night cycle with lights on at 8:30pm in a temperature (22±1ºC) and humidity (55±5%) controlled room.

All mice were allowed free access to water and a maintenance diet containing chow diet (CD) or high fat diet (HFD, 22% fat and 0.15% cholesterol, abdiets, Weerden, The Netherlands) in a 12-hour light/dark cycle, with room temperature at 21±2 °C. All cages contained wood shavings, bedding and a cardboard tube for environmental enrichment.

During the postoperative period, pain was relieved by a subcutaneous administration of buprenorfine (0.05-0.1 mg/kg; Temgesic®, Slough, UK) if the day the surgery was performed and in the morning after the surgery.

**10. Sample size**

Based on previous data (5-7), we know that the average σ = 55 (unstable plaque incidence). According to the Lenth’s statistical power calculation method and two-sample t test, (8) using an α=0.05, true differences of means=0.75 and power of 80% follows: n = 10 animals per group.

**11. Allocating animals to experimental groups**

Sections were scored blinded by two independent, very experienced pathologists with little inter- and intra-variability.

**12. Experimental outcomes**

Primary outcome measures were analyzed: significant increase in systolic blood pressure 2-4 weeks after the ligation of the left renal artery.

Secondary outcomes: Significant shrinkage of the left kidney was observed as control for successful renal artery ligation; white blood cell counts in all models.

Tertiary outcome measures were analyzed: expression of no lesion, lesion (which stage), thrombus formation, in addition to their incidence within the left common carotid artery.

Quaternary outcome measures were analyzed: structural features (intima size, intima to media ratio, necrotic core size, fibrous cap thickness) and compositional features of the plaque (macrophage, smooth muscle cell and smooth muscle cell content in addition to presence of intraplaque haemorrhages).

**13. Statistical methods**

All data were represented as individual data points along with mean values. Statistical analysis was performed for the corrected Vulnerability Index (VI_c_ ) but not for the individual parameters. Statistical analysis was done with GraphPad Prism 5 (GraphPad Software, LaJolla, CA). Unpaired Student’s t-test or Mann-Whitney (one variable) or one-way ANOVA with Bonferroni’s Multiple Comparison test (> 2 variables) were applied, as appropriate. p-values < 0.05 were considered as being statistically significant.

**RESULTS**

**14. Baseline data**

General monitoring of animal welfare: i.e. weight, appetite, behavioural characteristics (e.g. way of moving, isolation), physical characteristics (i.e., nose, mouth, eyes, skin, hair, eyes, posture), breathing, litter birth and size.

Daily, the behaviour (movement, posture) and the fur (care, any wounds due to fighting) were observed. Prior to the surgery, the animals were weighed to establish a reference value, after which animals were weighted until they reached the initial weight.

Intraoperative monitoring: Anaesthetized animals were monitored during the procedure to assure they stay in the proper anaesthetic plane (i.e. checking toe pinch reflex). Colour of mucous membranes and exposed tissues was checked for signs of incorrect oxygenation or blood perfusion (i.e. pink pale, dusky grey or blue). Respiratory pattern and frequency and body temperature were checked during the procedure.

Animals were excluded from the experiment, when they showed reduced or abnormal movement and behavior after the operation like do not eat (loss of weight >10% in two days compared to maximal bodyweight). For that reason the mice were weighted daily. If the animals showed signs of discomfort (hunched back, pilo erection, behavioral characteristics (e.g. way of moving, isolation), physical characteristics (e.g. nose, mouth, eyes, skin, hair, eyes, posture, breathing, litter and size) they would be euthanized.

**15. Numbers analyzed**

The health reports indicated no infection. All animals, excluded one in the group LCCA LRA HFD were analyzed. The excluded animal died during the surgery due to bleeding.

**16. Outcomes and estimations**

Taken together, we could observe for the performed models of atherosclerotic plaque vulnerability different characteristics summarized in Table 1. To note, all sections of different models were scored blinded by two independent, very experienced pathologists with little inter- and intra-variability.

|  | **LCCA LRA**  **CD** | **LCCA LRA HFD** | **LRA**  **Cast**  **CD** | **LRA**  **Cast**  **HFD** | **LCCA**  **Cast**  **CD** | **LCCA**  **Cast**  **HFD** | **Cast**  **HFD** |
| --- | --- | --- | --- | --- | --- | --- | --- |
| Technical  challenge | ••• | ••• | •• | •• | •• | •• | • |
| Duration | •• | •• | •• | •• | • | • | ••• |
| Non-Responders | •• | • | ••• | ••• | ••• | •• | •• |
| Robustness  (VI, VI_c_) | • | •• | • | • | • | ••• | ••• |
| Human-  like | • | • | - | • | • | •• | ••• |
| Incidence of |  |  |  |  |  |  |  |
| -mural  Thrombus | •• | - | • | •• | ••• | •• | - |
| -Lesion | ••• | ••• | • | • | • | ••• | ••• |
| -TFC/NC | •• | •• | - | • | • | •• | ••• |
| -IPH | ••• | ••• | • | - | ••• | ••• | - |

• low •• medium ••• high - n/a

**Table 1. Summary of features of the plaque destabilization models.** LCCA: left common coronary artery; LRA: left renal artery; CD: chow diet; HFD: high fat diet; VI: Vulnerability-Index; VI_c_: corrected Vulnerability-Index; FC: fibrous cap; NC: necrotic core; IPH: intraplaque hemorrhage.

**17. Adverse events**

Except for one mouse, no complications were observed. One mouse died because of surgical problems due to internal bleeding and was directly sacrificed. Monitoring of the mice during and after the operation showed fast recovery (e.g. stable temperature and no internal bleeding during he surgery; fast awakening, normal behaviour and fast gaining of same body weight as before the surgery).

**DISCUSSION**

**18. Interpretation/scientific implications**

The aim of our study was to compare different models of atherosclerotic plaque destabilization based on surgical approaches in combination with hypercholesterolemia. Hence, we performed models based on the combined partial ligation of LCCA and LRA, the combination of cast deployment around LCCA and partial ligation of LRA, the partial ligation of LCCA in combination with the cast placement around the LCCA and the cast placement around the LCCA. (LCCA: left common carotid artery; LRA: left renal artery) We determined the degree of the plaque vulnerability and the incidence of the specific stages of the atherosclerotic lesions within the models. Collectively, we have observed that the mouse model based on the cast implantation under hypercholesterolemia exhibited increased incidence of human-like atherosclerotic vulnerable plaques with less variability between the analyzed specimens

The comparison of further characteristics of the models (e.g. the discomfort for the animals, the duration of the experiments) we determined as important factors (summarized in Table 1). Based on these criteria we provide a possibility to select a model dependent of the research question that is going to be addressed and may lead to reduction of experimental animals.

**19. Generalisability/translation**

The vast use of mouse models to study atherogenesis has resulted in tremendous progress in the identification of novel molecular targets for treatment of early atherosclerosis. However its clinical application has been restricted since patients are not usually treated during early phases of the disease. On the other hand, mouse models of atherosclerotic plaque destabilization may help to identify molecular mechanisms involved in advanced atherosclerosis development that could be more easily translated to the clinical practice.

**20. Funding**

The research was supported by the Nederlandse Organisatie voor Wetenschappelijk Onderzoek (VIDI project 91712303), the Deutsche Forschungsgemeinschaft (DFG) (SO876/6-1, SFB1123 TP A06 and B05), and the LMU excellent program.

**References**

1. Naghavi M, Libby P, Falk E, Casscells SW, Litovsky S, Rumberger J, et al. From vulnerable plaque to vulnerable patient: a call for new definitions and risk assessment strategies: Part I. Circulation. 2003;108(14):1664-72.

2. Silvestre-Roig C, de Winther MP, Weber C, Daemen MJ, Lutgens E, Soehnlein O. Atherosclerotic Plaque Destabilization: Mechanisms, Models, and Therapeutic Strategies. Circulation Research. 2014;114(1):214-26.

3. Schwartz SM, Galis ZS, Rosenfeld ME, Falk E. Plaque rupture in humans and mice. Arteriosclerosis, thrombosis, and vascular biology. 2007;27(4):705-13.

4. Hewing B, Fisher EA. Preclinical mouse models and methods for the discovery of the causes and treatments of atherosclerosis. Expert opinion on drug discovery. 2012;7(3):207-16.

5. Sasaki T, Kuzuya M, Nakamura K, Cheng XW, Shibata T, Sato K, et al. A simple method of plaque rupture induction in apolipoprotein E-deficient mice. Arteriosclerosis, thrombosis, and vascular biology. 2006;26(6):1304-9.

6. Jin SX, Shen LH, Nie P, Yuan W, Hu LH, Li DD, et al. Endogenous renovascular hypertension combined with low shear stress induces plaque rupture in apolipoprotein E-deficient mice. Arteriosclerosis, thrombosis, and vascular biology. 2012;32(10):2372-9.

7. Cheng C, Tempel D, van Haperen R, van der Baan A, Grosveld F, Daemen MJ, et al. Atherosclerotic lesion size and vulnerability are determined by patterns of fluid shear stress. Circulation. 2006;113(23):2744-53.

8. Lenth RV. Statistical power calculations. Journal of animal science. 2007;85(13 Suppl):E24-9.
