# Supplementary material for: Atherosclerotic Plaque Destabilization in Mice: A Comparative Study
Source: PLoS One. 2015 Oct 22;10(10):e0141019. doi: 10.1371/journal.pone.0141019 (PMC4619621; doi:10.1371/journal.pone.0141019)
Supplement: S1 File — (DOC) [file pone.0141019.s002.doc]

**Supporting information**

**Atherosclerotic plaque destabilization in mice: A comparative study**

Helene Hartwig^1¶^, Carlos Silvestre-Roig^1,2¶^, Jeffrey Hendrikse^1^, Linda Beckers^3^, Nicole Paulin^2^, Kim Van der Heiden^4^, Quinte Braster^1^, Maik Drechsler^1,2^, Mat J. Daemen^1^, Esther Lutgens^2,3^, Oliver Soehnlein^1,2,5*^

^1^Department of Pathology, Academic Medical Center, Amsterdam, The Netherlands.

^2^Institute for Cardiovascular Prevention (IPEK), LMU Munich, Germany.

^3^Department of Medical Biochemistry, Academic Medical Center, Amsterdam, The Netherlands.

^4^Department of Cardiology, Biomedical Engineering, Erasmus MC, Rotterdam, The Netherlands.

^5^German Centre for Cardiovascular Research (DZHK), Munich Heart Alliance, Munich, Germany.

*These authors contributed equally to this work.

* Corresponding author

E-mail: [oliver.soehnlein@gmail.com](mailto:oliver.soehnlein@gmail.com)

^¶^These authors contributed equally to this work.

**Table A. Detailed description of models presented in original reports and modifications in this study.**

|  | **Original** | **Modification** | **Reference** |
| --- | --- | --- | --- |
| **LCCA LRA CD/HFD** |  |  | Jin,  2012 |
| Mice | *Apoe^-/-^*  age: 8 weeks  sex matched groups  CD  n=3-10 | males  CD or HFD  n=10 |  |
| Duration  (ligation until sacrifice) | 2, 4, 8 weeks | 4 weeks  start HFD 2 weeks before ligation |  |
| Anesthetic | intraperitoneal pentobarbital sodium | mask inhalation of isoflurane |  |
| LCCA Ligation | ligation (6-0 silk) of all LCCA branches except LSTA | ligation (7-0 silk) of all LCCA branches except LSTA |  |
| Sham LCCA Ligation | suture placement without ligation |  |  |
| LRA Ligation | left renal artery was tied off (6-0 silk) along with a spacer (diameter 0.11mm); spacer subsequently pulled out |  |  |
| Sham LRA Ligation | suture placement without ligation |  |  |
| Further treatment | angiotensin-II injection | none |  |

Model based on the combined partial ligation of LCCA and LRA CD or HFD (Jin et al., 2012). LCCA: left common carotid artery; LSTA: left superior thyroid artery; LRA: left renal artery; CD: chow diet; HFD: high fat diet.

**Table B. Detailed description of models presented in original reports and modifications in this study.**

|  | **Original** | **Modification** | **Reference** |
| --- | --- | --- | --- |
| **LRA Cast CD/HFD** |  |  | Jin,  2012 |
| Mice | *Apoe^-/-^*  age: 8 weeks  sex matched groups  CD  n=3-10 | males  CD or HFD  n=10 |  |
| Duration  (ligation until sacrifice) | 2, 4, 8 weeks | 8 weeks  start HFD 2 weeks before ligation |  |
| Anesthetic | intraperitoneal pentobarbital sodium | mask inhalation of isoflurane |  |
| LCCA Ligation | ligation (6-0 silk) of all LCCA branches except LSTA | cast placement around LCCA (constructive diameter 0.2mm) |  |
| Sham LCCA Ligation | suture placement without ligation | see Cast HFD model |  |
| LRA Ligation | left renal artery was tied off (6-0 silk) along with a spacer (diameter 0.11mm); spacer subsequently pulled out |  |  |
| Sham LRA Ligation | suture placement without ligation |  |  |
| Further treatment | angiotensin-II injection | none |  |

Model based on the combination of cast placement around LCCA and partial ligation of LRA CD or HFD (Jin et al., 2012). LCCA: left common carotid artery; LSTA: left superior thyroid artery; LRA: left renal artery; CD: chow diet; HFD: high fat diet.

**Table C. Detailed description of models presented in original reports and modifications in this study.**

|  | **Original** | **Modification** | **Reference** |
| --- | --- | --- | --- |
| **LCCA Cast CD/HFD** |  |  | Sasaki,  2006 |
| Mice | *Apoe^-/-^*  age: 8 weeks  sex male  CD  n=3-10 | female  CD or HFD  n=10 |  |
| Duration  (ligation until sacrifice) | Ligation-4 weeks-Sacr.  Ligation-4 weeks-Cuff-2days-Sacr.  Ligation-4 weeks-Cuff-4 days-Sacr. | Ligation-4 weeks-Cast-4 days-Sacr.  start HFD 2 weeks before ligation |  |
| Anesthetic | intraperetoneal pentobarbital sodium | mask inhalation of isoflurane |  |
| LCCA Ligation | ligation (6-0 silk) of all LCCA branches except LSTA | ligation (7-0 silk) of all LCCA branches except LSTA |  |
| Sham LCCA Ligation | suture placement without ligation |  |  |
| Cuff placement | polyethylene cuff (length 2mm; inside and outside diameter 0.580mm and 0.965mm) placement underneath ligation | cast placement around LCCA (constructive diameter 0.2mm) |  |
| Sham Cuff placement | suture placement without ligation |  |  |
| Further treatment | none |  |  |

Model based on the partial ligation of LCCA in combination with the cast placement around the LCCA under CD or HFD regime (Sasaki et al., 2006). LCCA: left common carotid artery; LSTA: left superior thyroid artery; LRA: left renal artery; CD: chow diet; HFD: high fat diet; Sacr.: Sacrifice.

**Table D. Detailed description of models presented in original reports and modifications in this study.**

|  | **Original** | **Modification** | **Reference** |
| --- | --- | --- | --- |
| **Cast HFD** |  |  | Cheng,  2006 |
| Mice | *Apoe^-/-^*  age: 15-20 weeks  sex male  HFD  n=5-9 | 8 weeks  female  HFD  n=10 |  |
| Duration  (cast until sacrifice) | 2 weeks HFD- Cast  -6 weeks  -9 weeks  -12 weeks | 2 weeks HFD- Cast  -9 weeks |  |
| Anesthetic | mask inhalation of isoflurane |  |  |
| Cast placement | cast placement around RCCA (variable constructive diameters) | cast placement around RCCA (constructive diameters 0.2mm) |  |
| Sham Cast placement | Control-Casts with non-constrictive diameter | suture placement without ligation |  |
| Further treatment | angiotensin-II minipumps | none |  |

Model based on cast placement around the LCCA (Cheng et al., 2006). LCCA: left common carotid artery; RCCA: right common carotid artery; LRA: left renal artery; CD: chow diet; HFD: high fat diet.

**Table E. Lesion classification.**

|  | **Histological**  **Classification** | **Description** |
| --- | --- | --- |
| **early** | Intimal thickening | Accumulation of SMC in intima  Absence of lipids or macrophage foam cells in intima |
| **advanced** | type I | Multiple layers of macrophage foam cells |
|  | type II | Thin Fibrous Cap Atheroma (TFCA)  Multiple layers of macrophage foam cells  Well-formed NC covered by thin FC  Eventually IPH |
|  | type III  Neointimal thickening | Highly increased intimal thickening  High amount of SMC and macrophages content  Partly NC formation |
| **complicated** | Thrombus | mural Thrombus |

Sections were scored using the main elements of Virmani’s histopathological classification (Virmani et al., 2000). SMC: smooth muscle cells, TFCA: thin fibrous cap atheroma; NC: necrotic core, FC: fibrous cap, IPH: intraplaque hemorrhage.

**
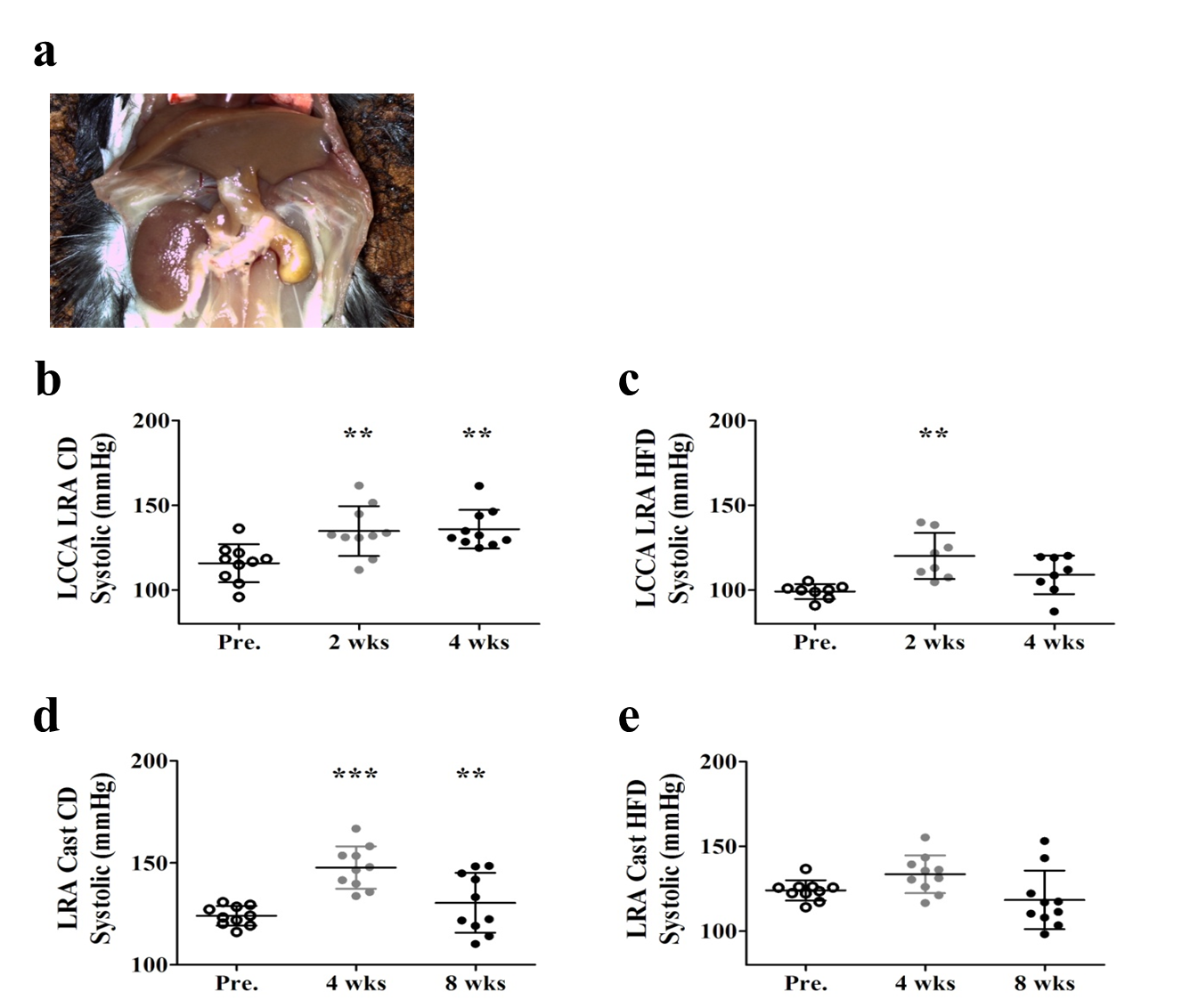
**

**Figure A. Control for successful induction of hypertension after ligation of the LRA.** (**a**) Significant shrinkage of the left kidney was observed in addition to significant increase in systolic blood pressure 2-4 weeks after the ligation to the measurement two weeks before the ligation (Pre.) (**b-d**). No significant difference could be detected in LRA Cast HFD (**e**). Graphs represent means ± SD (n=8-10; **p<0.01 with 1-way ANOVA with Bonferroni’s Multiple Comparison test). LCCA: left common carotid artery; LRA: left renal artery; CD: chow diet; HFD: high fat diet; Pre.: measurement before surgery.


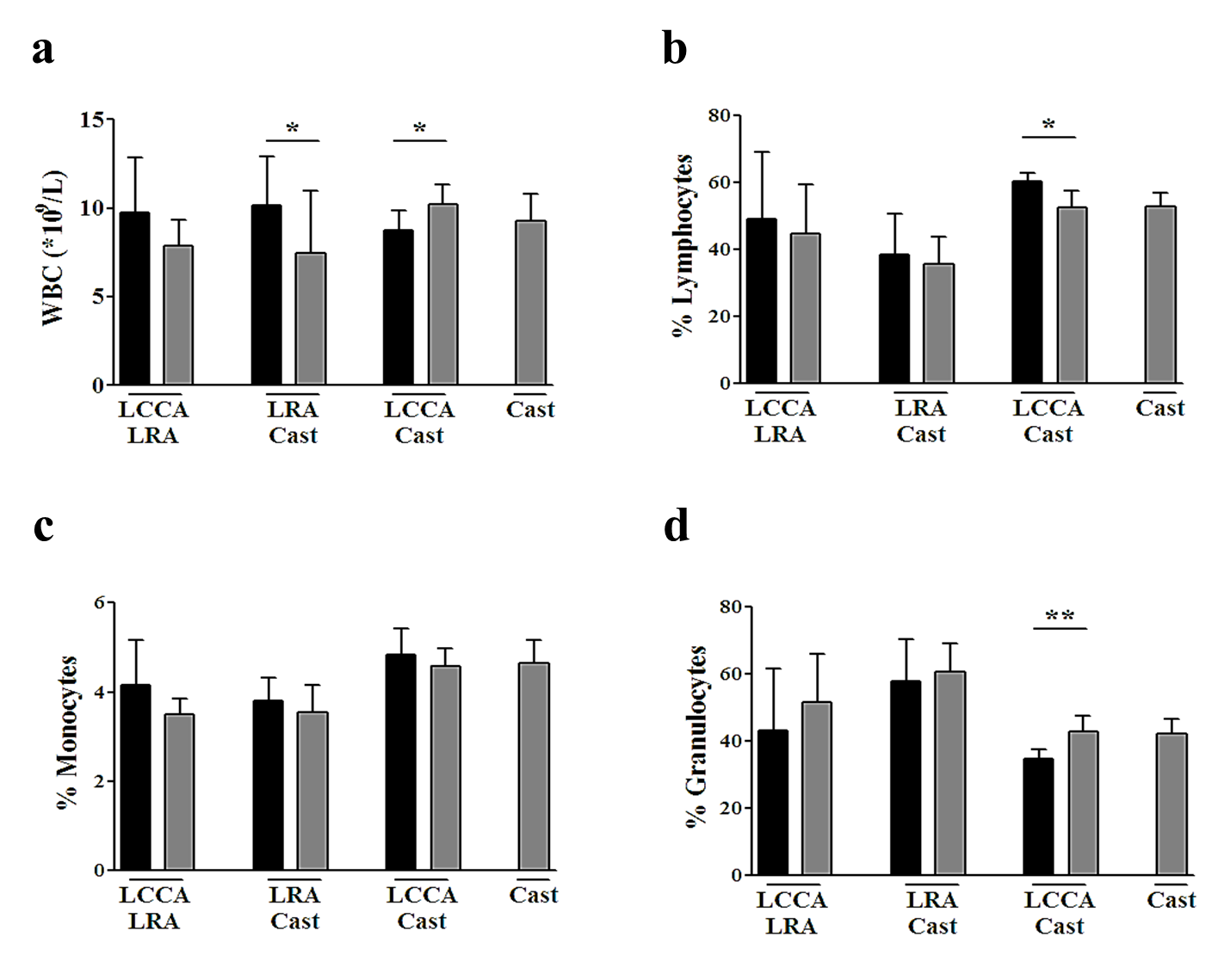


**Figure B. Differential white blood cell counts in all mouse models** including (**a**) WBC, (**b**) lymphocytes, (**c**) monocytes, (**d**) granulocytes. Significance for WBC was observed for LRA Cast (CD 10.15± 2.8% vs HFD 7.5± 3.5%) and LCCA Cast (CD 8.7± 1.1% vs HFD 10.2±1.1%). Furthermore a significant change in LCCA Cast was present in lymphocytes (**b**) (CD 60.5± 2.5% vs HFD 52.5±4.9%) and (**d**) granulocytes (CD 34.7±2.8% vs HFD 42.9±4.7%). Graphs represent means ±SD. n=3-10; *p<0.05 and **p<0.01 with 2-tailed *t* test. WBC: white blood cells; LCCA: left common carotid artery; LRA: left renal artery; CD: chow diet; HFD: high fat diet.


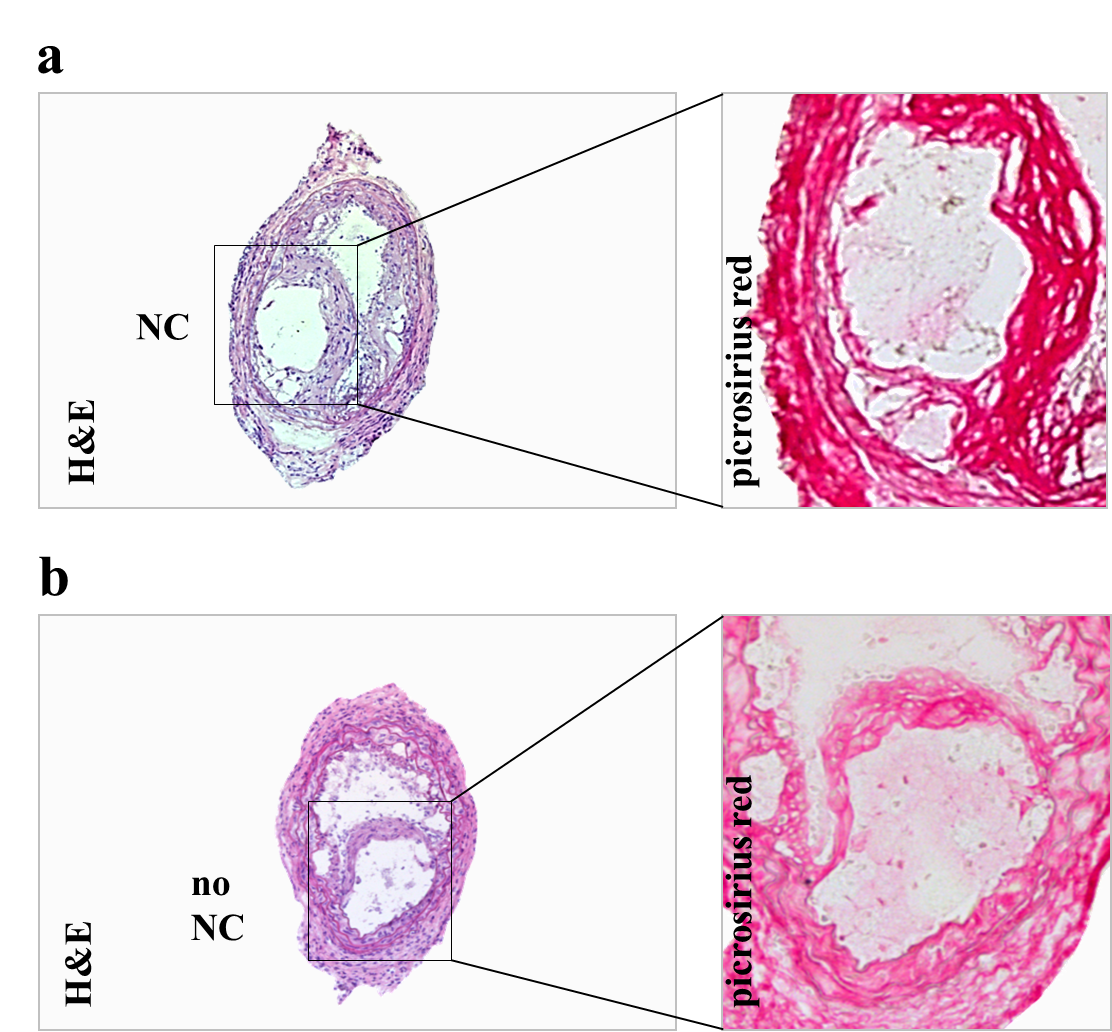


**Figure C. Necrotic core assessment.** (**a**) NC was defined as area absent of intact cells and deposition of cholesterol crystals underneath a formed FC. (**b**) False NC by tissue loss was observed in H&E. Assessment of NC was performed by additional control in accordance of further staining. NC: necrotic core; FC: fibrous cap.


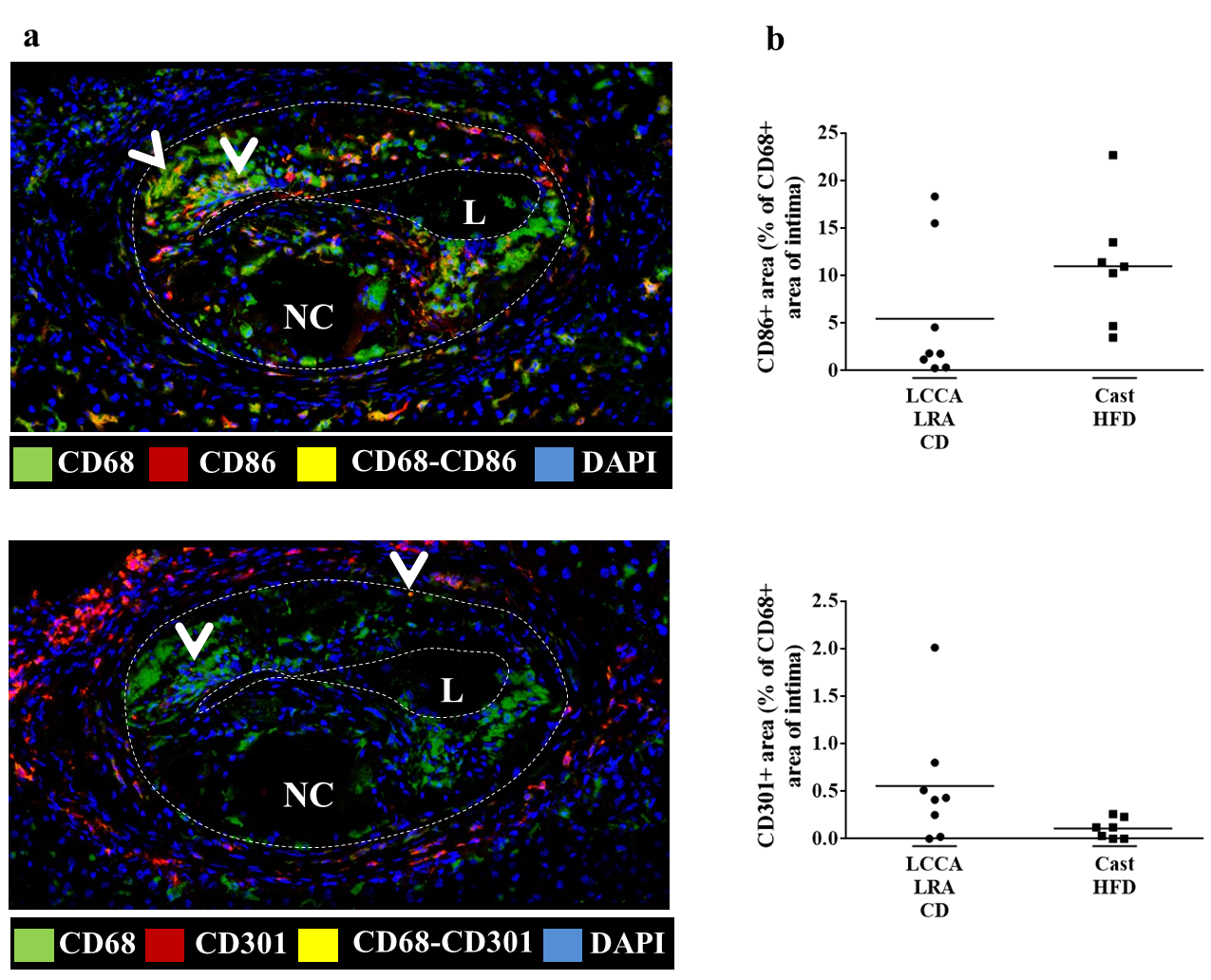


**Figure D. Characterization of the M1 and M2 macrophage subsets in the vulnerable atherosclerotic lesion.** (**a**) Double confocal immunofluorescence of atherosclerotic lesions showing CD68 (macrophage) and CD86 (M1 marker) on top or CD86 and CD301 (M2 marker) in the bottom. Arrows point double positive cells for CD68 and CD86 or CD301. (**b**) Quantification of lesional CD68-CD86 (top) or CD68-CD301 (bottom) area in the models (n=7-8; with 2-tailed *t* test). Cast HFD and LCCA LRA CD. LCCA: left common carotid artery; LRA: left renal artery; CD: chow diet; HFD: high fat diet.

**
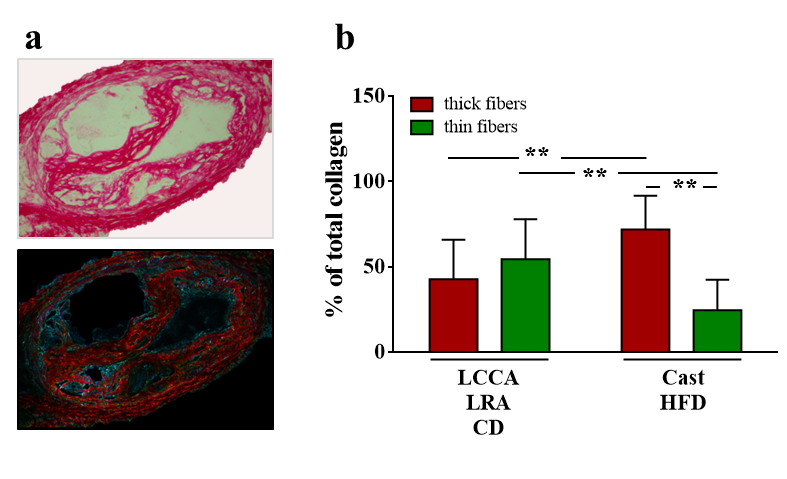
**

**Figure E. Collagen fiber composition.** (**a**) Representative images of picrosirius red-stained plaques visualized using polychromatic light (top) or polarized light illumination (bottom). (**b**) Analysis of collagen composition (red: thick fibers, green: thin fibers) in LCCA LRA CD and Cast HFD. Graphs represent means ± SD (n=7-8; **p<0.01 with 2-tailed *t* test). LCCA: left common carotid artery; LRA: left renal artery; CD: chow diet; HFD: high fat diet.


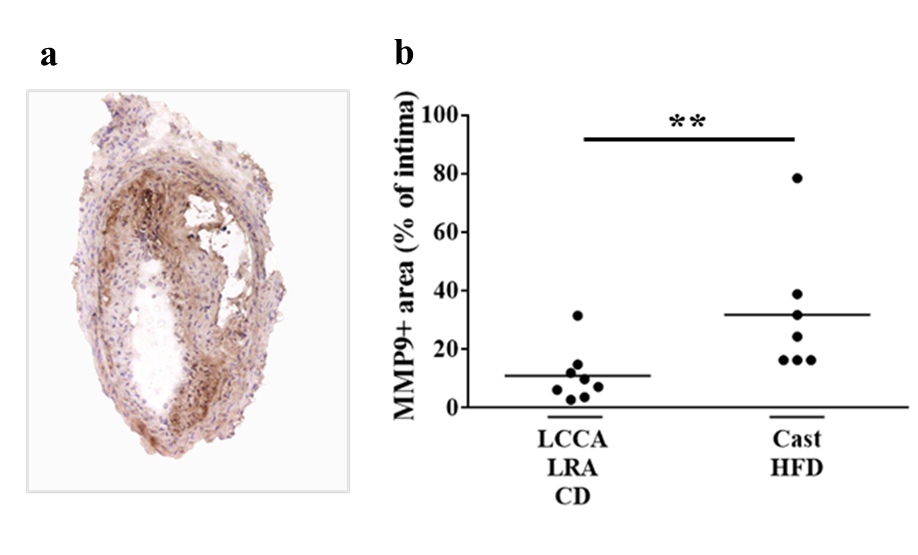


**Figure F. Analysis of lesional MMP9 expression.** (**a**) Representative image of MMP9 immunohistochemistry of a vulnerable atherosclerotic plaque (brown). (**b**) Quantification of lesional MMP9 positive area in models LCCA LRA CD and Cast HFD. Graphs represent means ± SD (n=7-8; **p<0.01 with 2-tailed *t* test). LCCA: left common carotid artery; LRA: left renal artery; CD: chow diet; HFD: high fat diet.


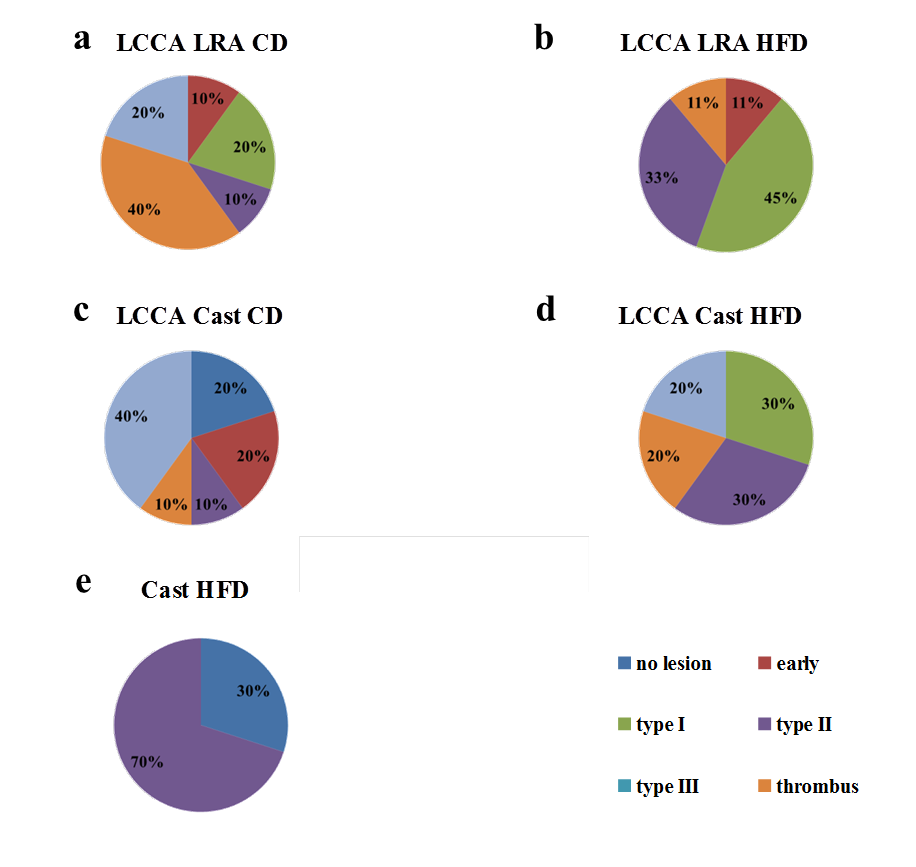


**Figure G. Relative distribution of lesion stages in models of plaque destabilization** (**a**) Model based on the combined partial ligation of LCCA and LRA fed CD or (**b**) HFD. (**c**) Model based on the partial ligation of LCCA in combination with the cast placement around the LCCA fed CD or (**d**) HFD. (**e**) Model based on cast placement around the LCCA fed HFD. LCCA: left common carotid artery; LRA: left renal artery; CD: chow diet; HFD: high fat diet.

**References**

**Cheng, C., Tempel, D., van Haperen, R., van der Baan, A., Grosveld, F., Daemen, M. J. A. P., Krams, R. and de Crom, R.** (2006). Atherosclerotic Lesion Size and Vulnerability Are Determined by Patterns of Fluid Shear Stress. *Circulation* **113**, 2744-2753.

**Jin, S.-x., Shen, L.-h., Nie, P., Yuan, W., Hu, L.-h., Li, D.-d., Chen, X.-j., Zhang, X.-k. and He, B.** (2012). Endogenous Renovascular Hypertension Combined With Low Shear Stress Induces Plaque Rupture in Apolipoprotein E–Deficient Mice. *Arteriosclerosis, Thrombosis, and Vascular Biology* **32**, 2372-2379.

**Sasaki, T., Kuzuya, M., Nakamura, K., Cheng, X. W., Shibata, T., Sato, K. and Iguchi, A.** (2006). A Simple Method of Plaque Rupture Induction in Apolipoprotein E–Deficient Mice. *Arteriosclerosis, Thrombosis, and Vascular Biology* **26**, 1304-1309.

**Virmani, R., Kolodgie, F. D., Burke, A. P., Farb, A. and Schwartz, S. M.** (2000). Lessons From Sudden Coronary Death: A Comprehensive Morphological Classification Scheme for Atherosclerotic Lesions. *Arteriosclerosis, Thrombosis, and Vascular Biology* **20**, 1262-1275.
